# Clinical Safety of Combined Targeted and *Viscum album* L. Therapy in Oncological Patients

**DOI:** 10.3390/medicines5030100

**Published:** 2018-09-06

**Authors:** Anja Thronicke, Shiao Li Oei, Antje Merkle, Harald Matthes, Friedemann Schad

**Affiliations:** 1 Network Oncology, Research Institute Havelhöhe, Kladower Damm 221, 14089 Berlin, Germany; anja.thronicke@havelhoehe.de (A.T.); shiaoli.oei@havelhoehe.de (S.L.O.); antje.merkle@havelhoehe.de (A.M.); harald.matthes@havelhoehe.de (H.M.); 2 Oncological Centre, Hospital Havelhoehe, Kladower Damm 221, 14089 Berlin, Germany; 3 Medical Clinic for Gastroenterology, Infectiology and Rheumatology CBF and Institute of Social Medicine, Epidemiology and Health Economics CCM, Charité University Hospital Berlin, 10117 Berlin, Germany

**Keywords:** safety analysis, toxicity profile, combinational therapy, targeted therapy, monoclonal antibody therapy, immune checkpoint inhibitors, treatment discontinuation, *Viscum album* L.

## Abstract

**Background**: Despite improvement of tumor response rates, targeted therapy may induce toxicities in cancer patients. Recent studies indicate amelioration of adverse events (AEs) by add-on mistletoe (*Viscum album* L., VA) in standard oncological treatment. The primary objective of this multicenter observational study was to determine the safety profile of targeted and add-on VA therapy compared to targeted therapy alone. **Methods**: Demographic and medical data were retrieved from the Network Oncology registry. Allocation to either control (targeted therapy) or combinational group (targeted/add-on VA) was performed. Safety-associated variables were evaluated by adjusted multivariable analyses. **Results**: The median age of the study population (*n* = 310) at first diagnosis was 59 years; 67.4% were female. In total, 126 patients (40.6%) were in the control and 184 patients (59.4%) in the combination group. Significant differences were observed between both groups with respect to overall AE frequency (χ^2^ = 4.1, *p* = 0.04) and to discontinuation of standard oncological treatment (χ^2^ = 4.8, *p* = 0.03) with lower rates in the combinational group (20.1%, 35% respectively) compared to control (30.2%, 60.5%, respectively). Addition of VA to targeted therapy significantly reduced the probability of oncological treatment discontinuation by 70% (Odds ratio (OR) 0.30, *p* = 0.02). **Conclusions**: Our results indicate a highly significant reduction of AE-induced treatment discontinuation in all-stage cancer patients when treated with VA in addition to targeted therapy.

## 1. Introduction

Targeted therapy composed of monoclonal antibodies (mAbs), tyrosine kinase inhibitors (TKIs), and immunotherapy (immune checkpoint inhibitors, ICIs) has been effectively utilized in cancer management [1,2,3]. However, meta-analyses reveal that not all targeted agents in oncological treatment are sufficient enough in terms of survival improvement [4,5] and that the effectiveness of even well-known approved targeted therapies for certain cancer subgroups remains controversial [5]. It is striking that only 35% of mAb indications and about 38% of TKI indications approved between 2009 and 2013 by the European Medicines Agency showed evidence of survival or healthrelated quality of life (HRQL) benefit over comparable treatment or placebo as shown by a systematic evaluation [6]. Similar results were published for these cancer treatments approved by the U.S. Food and Drug Administration [7]. It has repeatedly been reported that survival benefit of targeted therapy may come at the cost of toxicity potentiation when provided alone [8] or in combination with standard or chemotherapy [9,10,11,12,13,14,15,16,17]. A plethora of studies have been published documenting, besides good clinical outcome, potentiation of toxicities in combination treatment of chemotherapy or standard therapy with mAbs, targeted treatment, or immunotherapy [9,10,11,18]. Due to the current importance, protocols and guidelines for the management of immune-related adverse events (AEs) due to immunotherapy have recently been published [19,20,21,22,23,24,25]. In particular, cardiotoxicities and neurotoxicities, which are newly emerging immunotherapy-related AEs with relevant clinical impact, have been reported in the last years [26,27,28,29,30,31]. In Anthroposophic-integrative oncology it is a well-established concept to propose mistletoe (*Viscum album* L., VA) therapy concomitantly to antineoplastic treatment in cancer patients to improve besides HRQL the tolerability of oncology treatment-induced toxicity [32,33,34]. However, the underlying mechanisms of tolerability remain elusive. Recent results in all-stage cancer revealed that patients being treated with mAb therapy had a 5-fold higher probability of experiencing an AE compared to patients treated with additional VA [35]. Furthermore, add-on VA maintained ICI-induced toxicity profile in patients with advanced and metastasized lung cancer and melanoma [36,37]. Add-on VA therapy has been reported to entail a sound safety profile with no serious side effects [35,38,39,40]. We hypothesize that addition of VA therapy in the present study would not increase the known side effects of targeted treatment and might even reduce them.

## 2. Materials and Methods

### 2.1. Study Design

Safety of targeted therapy with or without concomitant VA extracts was examined in an observational study. The primary outcome of the study was to investigate the occurrence of adverse events during targeted therapy treatment with and without VA to assess the AE rate in oncological patients. The secondary outcome was the explorative analysis of factors that were associated with the risk of experiencing an AE and with discontinuation of treatment due to an AE.

### 2.2. Description of Study Participants

**Figure 1:**
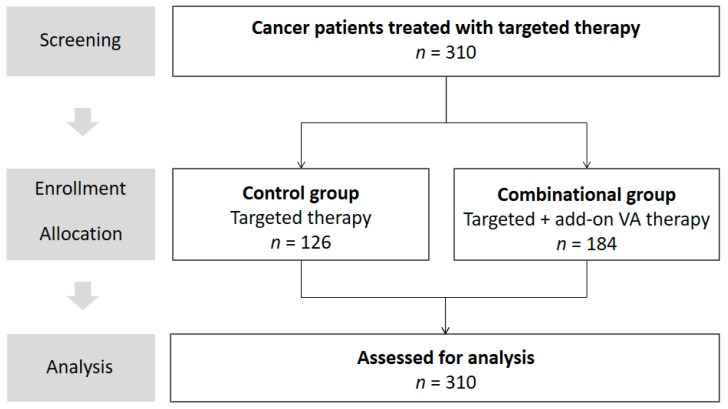
Flowchart of the study. VA, *Viscum album* L.

Oncological cancer patients consenting to be registered in the Network Oncology (NO), a certified German integrative-oncological clinical registry [41], were enrolled in the study between February 2010 and June 2017 (see flowchart, Figure 1). The following patients were included: patients who were 18 years or older (regardless of gender), who gave written consent and who received targeted therapy with or without concomitant Abnobaviscum^®^ (Pforzheim, Germany) VA therapy.

VA therapy was applied subcutaneously according to the summary of product characteristics (SmPC). Off-label intravenous or intralesional applications were provided in individual cases. The rationale for VA application in patients of the current study was the improvement of HRQL and self-regulation in cancer patients by meliorating cancer and therapy-related symptoms. VA was administered at the discretion of the physician. Patients having received a combination of targeted therapy and add-on VA therapy were allocated to the ‘combinational group’. The other patients having received targeted therapy without add-on VA were allocated to the ‘control group’. In addition, patients having received non-Abnoba VA ≥28 days after last day of targeted therapy or patients having received targeted therapy ≥28 days after last day of non-Abnoba VA therapy were allowed to be allocated to control group as well. Written informed consent was obtained. The Network Oncology (NO) registry study has been approved by the ethical committee of the Medical Association Berlin (Eth-27/10), date of approval: 04 February 2011.

### 2.3. Data Source and Assessment

Demographic data as well as information on diagnosis, co-morbidities and treatment information were retrieved from the NO registry. Aside from targeted therapy and VA therapy, information on applied chemotherapy was retrieved and analyzed. Cytostatic therapy included alkylating agents, alkaloids, anthracyclines, aminoglycosides, antimetabolites, antibiotic substances, folic acid analogs, nucleotide analogs and precursor analogs, platinum-based agents, vinca alkaloids and derivatives, taxanes, and topoisomerase inhibitors. AEs were designated according to International Council for Harmonisation of Technical Requirements for Pharmaceuticals for Human Use (ICH) guidelines topic E2A [42] and were defined as “any untoward medical occurrence in a patient or clinical investigation subject administered a pharmaceutical product and which does not necessarily have to have a causal relationship with this treatment”. In terms of severity, AEs were evaluated according to the Common Terminology Criteria for AEs (CTCAE) v4.03 [43] and designated as serious or non-serious according to ICH guidelines. AEs were classified as preferred terms according to the Medical Dictionary for Regulatory Activities (MedDRA^®^, Redwood City, CA, USA) Version 15.0 and grouped by System Organ Classes (SOC).

### 2.4. Statistical Methods

Univariate two-sided Fisher’s exact test or Chi-squared statistical analysis were performed to detect differences in AE rates and treatment discontinuation rates between groups. Multivariable regression analysis with binary outcome of experienced AE (yes/no) was performed to identify associated factors in the study group adjusting for age (in years), gender (male/female), tumor origin (breast, digestive/gastrointestinal, hematologic/blood, respiratory/thoracic and other including endocrine, neuroendocrine, eye, genitourinary, gynecologic, musculoskeletal, neurologic and skin tumors), targeted therapy (yes/no), add-on VA therapy (yes/no), surgery (yes/no), and radiation (yes/no). Multivariable regression analyses in a subgroup experiencing AE with each of the following binary outcomes discontinuation of treatment (yes/no), groups (targeted vs. combinational), therapies (each targeted therapy vs. all other targeted therapies e.g., bevacizumab vs. non-bevacizumab etc.), cancer entities (each cancer entity vs. all other cancer entities, e.g., gastrointestinal vs. other cancer etc.) were performed to identify associated factors in the study group adjusting for age (in years) and gender (male/female). If applicable, Brier scores as comparisons of predicted risks with observed outcomes at individual level where outcome values are either 0 or 1 were indicated [44]. Furthermore, Nagelkerke’s R2 values as percentages of variation of the outcome explained by the predictors in the model were indicated, if applicable [45].

Continuous variables were described as median with interquartile range (IQR), with categorical variables as frequencies and percentages. Data distributions were inspected graphically using box plots and histograms and were arithmetically examined for skewness. Stepwise backward variable selection with Akaike information criterion was performed for consideration of parameters within regression models. *p*-values < 0.05 were considered to be significant. All statistical analyses were performed using the software R (Version 3.3.0, R Development Core Team, Vienna, Austria) [46].

## 3. Results

### 3.1. Baseline Characteristics

In total, 310 patients were treated; of these 126 patients (40.6%) received targeted therapy without add-on VA therapy (control group). and 184 patients (59.4%) received targeted and add-on VA therapy (combinational group) between January 2010 and June 2017 (see flowchart, Figure 1).

Table 1 shows the baseline characteristics of the patients. In the total study group, 67.4% were female. The median age was 59 years (interquartile range, IQR: 49.0–68.0 years). The most prevalent cancers were breast cancer (36.5%), digestive/gastrointestinal cancer (27.1%), and respiratory/thoracic cancer (20%). Breast cancer patients made up almost half the proportion of the control group (46.8%), followed by patients with hematologic/blood cancer (23.0%), respiratory/thoracic cancer (15.1%), and digestive/gastrointestinal cancer (13.5%).

The highest proportion of patients in the combinational group were patients with digestive/gastrointestinal diseases (36.4%) followed by patients with breast cancer (29.4%) and respiratory/thoracic diseases (23.4%). As to differences between groups, the proportion of breast cancer patients (*p* = 0.002), of patients with digestive/gastrointestinal cancer (*p* < 0.001), and the proportion of patients with Union for International Cancer Control (UICC) stage IV cancers (*p* < 0.001) were significantly lower in the control compared to the combinational group. Hematological/blood cancers (*p* < 0.001) as well as cancers with unknown UICC stage (*p* = 0.007) were significantly more prevalent in the control compared to the combinational group.

### 3.2. Oncological Pharmacological and Non-Pharmacological Treatment

Cancer-related surgery was performed in 236 patients (76.1%) and radiation in 141 patients (45.6%) with almost balanced proportions of patients in both treatment groups, respectively (surgery: control group 74% vs. combinational group 78.3%; radiation: control group 49.2% vs. 35.9% in the combinational group), data not shown. Chemotherapy (CTx) was applied to 82 patients (26.5%), hereby with a significantly (*p* < 0.001) smaller proportion of patients in the control (12.7%) versus combinational group (35.9%), data not shown. As to targeted therapy, trastuzumab (34.5%), bevacizumab (24.5%), and rituximab (10.6%) were the most frequently applied monoclonal antibodies, whereas erlotinib (12.3%) was the most frequently applied TKI (Table 2).

A relevant higher proportion of patients in the combinational group received bevacizumab (35.3%) or erlotinib (19.0%) compared to the control group (8.7% and 2.4%, respectively, *p* < 0.001). In addition, the proportion of patients in the combinational group that received erlotinib was higher (19%) compared to the proportion of patients in the control group (2.4%, *p* < 0.001). In contrast, a significantly higher proportion of patients from the control group was treated with trastuzumab (48.4%) or rituximab (21.4%) compared to the combinational group (25% and 3.3%, respectively, *p* < 0.001). Targeted therapy lasted in median 3.1 months (93 days) (IQR 1.6–6.2 months).

As to add-on VA treatment in the combinational group VA fraxini (63.6%), VA quercus (17.6%) and VA mali remedies (10.7%) were most frequently applied (Table 3). The duration of add-on VA therapy was in median 3.8 months (114 days) (IQR 1.1–11.9 months).

### 3.3. Adverse Events Related to Targeted and to Combinational Treatment

The total AE-frequency in the current study group was 11% with 34 of a total of 310 patients (Table 4). With respect to treatment groups, 38 AEs (30.2%) were experienced by 17 (13.5%) patients in the control and 37 AEs (19.8%) were experienced by 17 (9.2%) patients in the combinational group. The AE frequency significantly differed between both groups (χ^2^ = 4.12, *p*-value = 0.042). The most often reported AEs in the total study cohort were anemia, nausea, vomiting, and pyrexia (Table 4). The most frequent AEs observed in patients of the control group were anemia, pain, nausea, and vomiting (2.4% each), while in the combinational group pyrexia and reduced general conditions (2.2% each) were reported most often. In terms of System Organ Class (SOC) most AEs were of the category ‘blood and lymphatic system disorder’ (5.5%), closely followed by ‘general disorders and disorders of the administration site’ (4.2%) and ‘gastrointestinal disorders’ (2.9%, Table 4).

**Figure 2:**
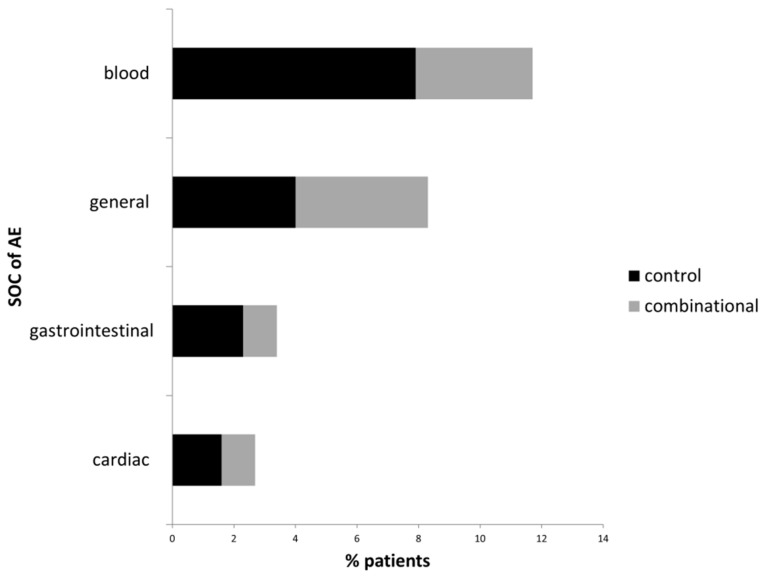
Most frequently (≥2% of patients per group) reported AEs classified by SOC. Black bars, % patients of the control group experiencing an AE; grey bars, % of patients of the combinational group experiencing an AE; SOC: System Organ Class.

As to treatment group differences, the proportion of patients reporting ‘general disorders and disorders of the administration site’ was almost equal in both treatment groups (6.4% control vs. 6.5% combinational group, Figure 2). The proportion of patients reporting psychiatric disorders (‘reduced general condition’) was higher in the combinational (2.2%) than in the control group (0.8%), however, this difference was not significant. For all other SOCs, the patient’s proportion was higher in the control group (Figure 2). No serious AEs and serious adverse reactions (ICH) [40] were documented for the total study cohort. No deaths from study-drug toxic effects were reported.

### 3.4. Factors Associated with Occurrence of AE

Multivariable logistic regression analysis adjusting for demographic, treatment-related and tumor location-related variables revealed that breast cancer was associated with a highly statistically significant 87% reduced probability to report an AE (Odds ratio (OR) 0.13, 95% confidence interval (CI): 0.04–0.40, *p* < 0.001), data not shown. With regard to standard oncological treatment, radiation increased the AE probability by factor 1.9 with a tendency towards significance (OR 1.93, 95%CI: 0.92–4.05, *p* = 0.08). For the outcome ‘AE’ no other significant prediction variable was observed; however, an increased (not significant) probability in the control group (OR 1.840, 95%CI: 0.90–3.77, *p* = 0.1) and a reduced (not significant) probability for experiencing an AE in the combinational group (OR 0.54, 95%CI: 0.27–1.12, *p* = 0.1) were observed.

### 3.5. Treatment Discontinuation in Patients Experiencing Adverse Events

**Figure 3:**
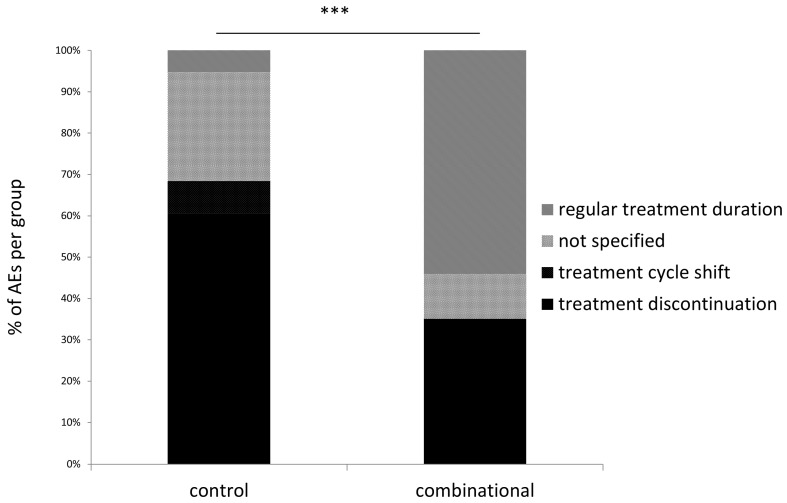
Status of treatment discontinuation. ***, *p*-value < 0.001.

Of 34 patients that experienced 75 AEs, in 15 patients (44.1% of patients with AEs) 36 standard oncological treatments were discontinued (48% of all AEs), 3 treatment cycles (4% of all AEs) were delayed (treatment cycle shift), and 22 treatments were regularly continued (29.3% of all AEs, Figure 3). In those patients experiencing AEs, differences with regard to treatment discontinuation and changes were significant between treatment groups (*p* < 0.001, Figure 3). In detail, 23 (60.5%) treatments were discontinued in the control group compared to 13 (35%) treatment discontinuations in the combinational group (χ^2^ = 4.84, *p* = 0.03). Only two (5.3%) treatments in the control group had a regular duration compared to 20 (54.1%) regular treatments in the combinational group (χ^2^ = 21.5, *p* < 0.001). Treatment cycle delays due to AEs were noted only in the control group (*n* = 3, 7.9%) while none were noted in the combinational group (*p* > 0.05, not significant). For 10 (26.3%) AEs in the control group, no specifications regarding treatment delays or discontinuations were stated compared to 4 (10.8%) in the combinational group (*p* > 0.05, not significant).

### 3.6. Factors Associated with Oncological Treatment Discontinuation

**Figure 4:**
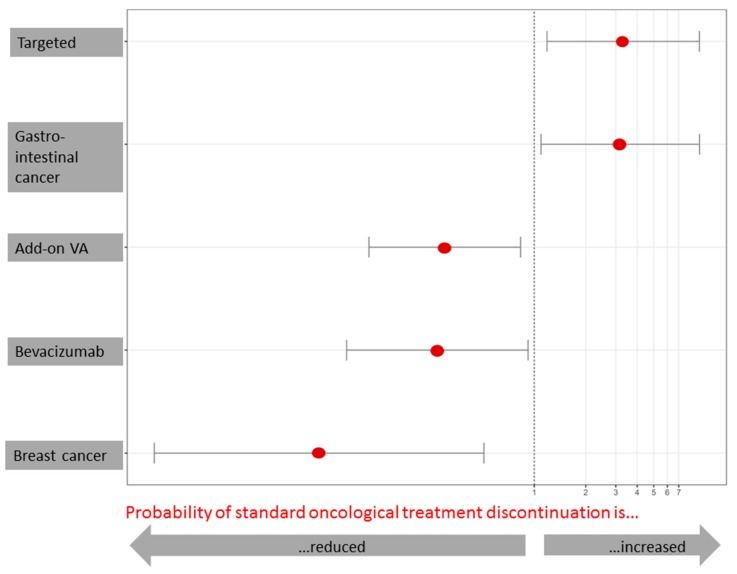
Probability of oncological treatment discontinuation in the AE-experiencing subgroup (*n* = 34). Adjusted multivariable logistic regression analysis. Factors presented are statistically significant (*p* < 0.05) for reduced probability (left-hand side from the indicated margin) or for increased probability (right -hand side from the indicated margin) of oncological treatment discontinuation due to adverse side effects, i.e., three times higher probability of discontinuation of standard oncological therapy when applying targeted compared to combined therapy, three times higher probability of discontinuation of standard oncological therapy in gastrointestinal tumors compared to non-gastrointestinal tumors, 70% reduced risk of discontinuation of standard oncological therapy when applying add-on VA therapy compared to targeted therapy without add-on VA therapy, 73% reduced risk of discontinuation of standard oncological therapy when applying bevacizumab compared to therapy without bevacizumab, 94% reduced risk of discontinuation of standard oncological therapy in breast cancer compared to non-breast cancer.

Adjusted multivariable regression analysis revealed that combinational therapy including add-on VA therapy significantly reduced the probability of oncological treatment discontinuation (OR 0.302, 95%CI: 0.108–0.832, *p* = 0.02) whereas targeted therapy without add-on VA therapy significantly increased the probability (3.314, 95%CI: 1.19–9.25, *p* = 0.02) in patients experiencing AEs (Figure 4). However, a reduced discontinuation of treatment was also associated with bevacizumab therapy compared to all other targeted therapies (OR 0.272, 95%CI: 0.080–0.920, *p* = 0.036, Figure 4). Gastrointestinal cancer compared to all other cancer was significantly associated with an increased risk (OR 3.18, 95%CI: 1.10–9.25, *p* = 0.03) while breast cancer was associated with a reduced risk of treatment discontinuation (OR 0.06, 95%CI: 0.01–0.51, *p* = 0.01). The analysis model revealed an accuracy of risk prediction (Brier scores) between 0.22–0.23. The Nagelkerke’s R2 values were 0.11 for the analysis with the predictive variables ‘targeted therapy vs. targeted plus add-on VA therapy’ and ‘bevacizumab vs. other targeted’ indicating a medium effect ($f=\frac{\sqrt{0.121}}{1-0.121}=0.53$) according to Cohen. The Nagelkerke’s R2 value was 0.20 for the predictive value breast vs. other cancer which accordingly corresponded to a high effect (f = 0.82).

## 4. Discussion

Amelioration of adverse mAb-related side effects [35], maintenance [36,37], and even the reduction of ICI-induced toxicity profile [47] by add-on VA has recently been acknowledged. In the present study we investigated, whether add-on VA may have similar beneficial effects in patients treated with targeted therapies including TKIs in addition to mAbs and ICIs. Our results confirmed this hypothesis indicating that the overall AE-frequency was significantly diminished in the combinational group (targeted therapy plus add-on VA) compared to control (targeted therapy without concomitant VA). In addition, the risk of treatment abortion due to an AE was significantly reduced in the combinational group compared to the control group confirmed by adjusted multivariable logistic regression analysis.

As to baseline differences, the combinational group was characterized by higher proportions of patients with gastrointestinal and lung tumors as well as of patients with tumors in a metastasized stage indicating the overall application areas of add-on VA treatment. We could recently show that advanced or metastasized stage colorectal cancer (mCRC) and also lung cancer rather than early stage carcinoma patients apply Integrative Oncology (IO) therapies including add-on VA therapy [48]. This may also explain the more frequent application of bevacizumab and erlotinib in the combinational group as these are approved standard therapies in unresectable mCRC and all stage non-small cell lung cancer, respectively [49,50]. It would as well explain the higher frequency of applied cetuximab in the combinational group for the therapy of refractory metastatic mCRC [51]. On the contrary, the significantly higher proportion of breast cancer patients in the control group associates with a more frequent application of trastuzumab being applied as an adjuvant treatment of human epidermal growth factor receptor (Her2)-overexpressing breast cancer [52]. Another significant proportion in the control group consists of patients with hematological cancers and the significantly higher application of rituximab may be attributable to the treatment of stage III–IV non-Hodgkin’s lymphoma and recurrent/refractory chronic lymphocytic leukemia [53]. Recent results of our group comparing combined mAb plus VA therapy versus overall mAb therapy without VA support the observation of the present study that patients of the control group were more likely breast cancer patients or patients with hematological cancers and that diagnosis of cancer occurred at earlier stages [35].

The results of the present study reveal that the overall AE-frequency was significantly diminished in the group where patients received a combination of targeted therapy and add-on VA extracts. This complies with earlier observations as to mAb treated patients [35] and ICI-treated patients [54]. However, for targeted therapies including mAbs, TKIs, and ICIs this has not yet been shown. The rate of patients experiencing an AE in the control group is lower than published AE rates of clinical studies involving similar medical agents [11,36,37]. However, the order of frequency and the types of AEs are in line with those published and most were expected according to clinical studies and SmPCs of targeted therapies and chemotherapy. The higher percentage of patients with cardiac adverse side effects in the control group (in the category ‘blood and lymphatic disorders’) may be explained by the fact that cardiac dysfunction is attributable to the Her2-targeted mAb trastuzumab, especially in combination with anthracyclines [47]. Cardiotoxicities are, among others, newly emerging ICI-related AEs reported in the last year [26,27,28,29,30,31]. Blood disorders were more frequently reported in the control group. As anemia is frequently reported as a result of applied chemotherapy and/or targeted therapy [18] it could explain the increased rate in the control group. In addition, thromboembolic events and hemorrhage were documented for bevacizumab in combination with CTx [10]. Gastrointestinal adverse events have been reported to be increased in patients during mAb, targeted, or ICI therapy [9,10,11,18] and may explain the higher percentage of these events in the control group. Accordingly, as to diarrhea the incidence was reported to be elevated in combined CTx/mAb [11] and CTx/targeted therapy [18] compared to CTx alone. General and infusion-related symptoms may be attributable to both targeted therapy including anti-endothelial growth factor receptor (EGFR) mAb therapy [55,56] and VA therapy [38,39] and may explain the equal distribution in both treatment groups of the present study. An increased incidence of skin toxicities among others were reported for combinational CTx/mAb compared to CTx alone [11]. For instance, a higher incidence of grade 3 or 4 rash (Risk ratio (RR) = 11.2) was observed after combined CTx/targeted therapy compared to a CTx in former studies [18]. Also, mild and moderate infusion-related and allergic skin reactions have been reported earlier for add-on VA treatment [35]. As pyrexia among others is one of the most frequently, and indeed desired, adverse events of mistletoe application [38] it may explain its higher frequency in the combinational group. We speculate that the higher rate of psychiatric disorders in the form of reduced general condition in the combinational group may be rather attributable to the general cancer-related state of the patients than to treatment-related side effects as a significantly higher proportion of combinational group patients were in stage IV at diagnosis.

We could show in the present study that the probability of standard oncological treatment discontinuation due to an AE in patients treated with targeted therapy was significantly reduced by 70% when additional VA was applied. Vice versa, the probability of experiencing a treatment discontinuation was 3-fold higher when treated with targeted therapy. This number complies with numbers of a previous mAb therapy study in which the probability of experiencing an AE was 5-fold higher in mAb treated patients compared to those treated concomitantly with VA [35]. We assume that the lower frequency of AEs and the lower rate of treatment discontinuations in the combinational group are attributed to the addition of VA to targeted therapy. This is in line with published tolerability results of studies analyzing the safety profile of mAb and ICI therapies without and with add-on VA therapy [35]. The specific underlying mechanisms of VA’s toxicity-ameliorating effects in the present study remain elusive, and the well-published VA-mediated immune-stimulatory [57,58], anti-proliferative [59,60,61,62], anti-angiogenic [63], and anti-invasive [64] effects may not sufficiently explain them. VA extracts have been shown to in vitro and in vivo improve DNA-repair of radiation-, ultraviolet radiation- and cyclophosphamide-induced damage of lymphocytes of breast cancer patients [65,66,67,68,69] and further in the gamma-ray damaged peripheral blood mononuclear cells of breast cancer patients [70] and this may serve as a hypothesis for the mechanism of the improvement in adverse events experienced by cancer patients. Furthermore, as short-term overall HRQL induced by additional VA-treatment has been reported [71,72,73] this may result in a shifted patient’s perception of a potential AE as being less burdensome.

Interestingly, bevacizumab compared to all other applied targeted therapies showed a reduced probability of standard oncological treatment discontinuation in the present study which complies with results from a meta-analysis evaluating add-on bevacizumab’s side effects as rather acceptable while significantly improving progression free survival and overall response rate [74]. Even though bevacizumab is mainly indicated as a targeted treatment in unresectable mCRC, the increased probability of treatment discontinuation in gastrointestinal cancer in the present cohort may rather be based on advanced cancer stages than on treatment toxicity profiles [48]. In fact, we could observe a reduced probability of treatment discontinuation in patients being diagnosed with breast cancer, which may reflect the ‘healthier’ early-stage cancer condition of this group as discussed earlier and the generally better-tolerated treatment. Patients with breast cancer are often skeptical and anxious towards standard oncological care [75]. However, as shown here, it may be reasonable to conclude for breast cancer patients that an increase of AEs or treatment discontinuation due to better-tolerated targeted therapy regiments are rather rare. As additional VA therapy leads to a lower discontinuation rate in targeted therapies as seen in our cohort, it may contribute to a higher effectiveness and thus be favorable for patients.

The drawn conclusions of the present results are limited due to the study’s non-longitudinal, non-randomized, and non-controlled nature possibly assessing heterogeneous groups of patients as to tumor entities, disease stage, and variable strengths and forms of application of concurrent therapies. Nevertheless, this multicenter study provides a first impression on the safety aspects of concomitant VA to targeted therapy treatment in oncological patients.

## 5. Conclusions

The results of the present study indicate that the AE frequency was significantly lower in patients treated with combined targeted and concomitant VA therapy compared to patients treated with targeted therapy alone. AEs experienced in the combinational group resulted in significantly less frequent standard oncological treatment abortions. Reduction of treatment discontinuations due to an AE are significantly associated with additional VA therapy in patients treated with targeted therapy as shown by adjusted multivariable regression analyses. Our observations are in line with published reduced AE rates in patients treated with oncological standard therapy and add-on VA. The probability for breast cancer patients to experience an AE or treatment discontinuation during targeted therapy is low, and add-on VA may positively influence this effect in cancer patients.

## Figures and Tables

**Table 1:** Characteristics of patients.

Patient Characteristics	Total Cohort	Control Group	Combinational Group	Significance
	*n* = 310	*n* = 126	*n* = 184	
Age at first diagnosis, years, median (IQR)	59.0 (49.0–68.0)	59.0 (48.0–67.0)	60.0 (50.0–69.0)	—
**Gender**				
Male, *n* (%)	95 (30.6)	33 (26.2)	62 (33.7)	—
Female, *n* (%)	215 (67.4)	93 (73.9)	122 (66.3)	—
**Cancer by body location, *n* (%)**				
Breast, *n* (%)	113 (36.5)	59 (46.8)	54 (29.4)	** ^a)^
Digestive/Gastrointestinal, *n* (%)	84 (27.1)	17 (13.5)	67 (36.4)	** ^b)^
Hematologic/Blood, *n* (%)	36 (11.6)	29 (23.0)	7 (3.8)	** ^b)^
Respiratory/Thoracic, *n* (%)	62 (20.0)	19 (15.1)	43 (23.4)	—
Genitourinary, *n* (%)	7 (2.3)	1 (0.8)	6 (3.3)	—
Gynecologic, *n* (%)	4 (1.3)	1 (0.8)	3 (1.63)	—
Musculoskeletal, *n* (%)	1 (0.3)	0	1 (0.5)	—
Skin, *n* (%)	3 (1.0)	0	3 (1.6)	—
**UICC stage at first treatment, *n* (%)**				
0, *n* (%)	1 (0.3)	0	1 (0.5)	—
I, *n* (%)	22 (7.1)	10 (7.9)	12 (6.5)	—
II, *n* (%)	43 (13.9)	22 (17.5)	21 (11.4)	—
III, *n* (%)	62 (20.0)	24 (19.0)	38 (20.7)	—
IV, *n* (%)	108 (34.8)	30 (23.8)	78 (42.4)	** ^b)^

Characteristics of the patients included in the study, total cohort and respective treatment groups; IQR, interquartile range; UICC, Union for International Cancer Control; %, as percent from total patient number *n* from each group; Significance codes ^a)^ *p* = 0.002, ^b)^ *p* < 0.001, “—” not statistically significant.

**Table 2:** Characterization of targeted therapy.

Target Therapy Class	Target	Total Cohort	Control Group	Combinational Group	Significance
		*n* = 310	*n* = 126	*n* = 184	
**Monoclonal antibodies**					
bevacizumab, *n* (%)	VEGFR	76 (24.5)	11 (8.7)	65 (35.3)	*** ^a)^
cetuximab, *n* (%)	EGFR	23 (7.4)	5 (4.0)	18 (9.8)	—
panitumumab, *n* (%)	EGFR	12 (3.9)	2 (1.6)	10 (5.4)	—
rituximab, *n* (%)	CD20	33 (10.6)	27 (21.4)	6 (3.3)	*** ^a)^
trastuzumab, *n* (%)	HER2	107 (34.5)	61 (48.4)	46 (25.0)	*** ^a)^
**Immunotherapy**					
ipilimumab, *n* (%)	CTLA-4	1 (0.3)	—	1 (0.5)	—
nivolumab, *n* (%)	PD-1	3 (1.0)	—	3 (1.6)	—
pembrolizumab, *n* (%)	PD-1	5 (1.6)	2 (1.6)	1 (0.5)	—
**Tyrosine kinase inhibitors**					
erlotinib, *n* (%)	EGFR	38 (12.3)	3 (2.4)	35 (19.0)	*** ^a)^
gefitinib, *n* (%)	EGFR	9 (2.9)	3 (2.4)	6 (3.3)	—
sorafenib, *n* (%)	Dual Raf-kinase/VEGFR	7 (2.3)	—	7 (3.8)	—
sunitinib, *n* (%)	Receptor tyrosine kinases	7 (2.3)	1 (0.8)	6 (3.3)	—

Most frequent applied targeted therapies in the control (targeted therapy) and combinational group (targeted + VA) with their respective molecular targets. *n*, number of patients; %, as percent from total patient number *n* from each group; Significance code: ^a)^ *p* ≤ 0.001, “—” not statistically significant; CTLA-4, cytotoxic T lymphocyte-associated antigen 4; CD20, B-lymphocyte antigen CD20; EGFR, endothelial growth factor receptor; HER2, human epidermal growth factor receptor 2; PD1, programmed cell death protein 1; Raf, rapidly accelerated fibrosarcoma; VEGFR, vascular endothelial growth factor receptor.

**Table 3:** Characterization of add-on VA therapy.

VA Remedy	Total	Colorectal Cancer	Lung Cancer	Breast Cancer	Other Cancer
	*n* (%)	*n* (%)	*n* (%)	*n* (%)	*n* (%)
VA	184	49 (100)	40 (100)	50 (100)	45 (100)
VA abietis	4 (2.1)	-	1 (2.0)	3 (6.0)	-
VA aceris	6 (3.2)	-	5 (10.2)	-	1 (2.2)
VA craetegi	2 (1.1)	1 (2.0)	-	-	1 (2.2)
VA fraxini	119 (63.6)	23 (46.9)	32 (65.3)	31 (62.0)	34 (75.6)
VA mali	20 (10.7)	2 (4.1)	1 (2.0)	16 (32.0)	1 (2.2)
VA quercus	33 (17.6)	27 (55.1)	2 (4.1)	-	4 (8.9)
VA pini	6 (3.2)	1 (2.0)	1 (2.0)	3 (6.0)	1 (2.2)

Characterization of add-on VA remedies in accordance to their respective host trees. Numbers in rows and columns do not necessarily add to 100%, as patients may have received various combinations of treatment. *n*, number; %, percent; -, not applicable.

**Table 4:** Adverse events per treatment group.

System Organ Class	Adverse Event	Control Group	Combinational Group
		*n* = 126	*n* = 184
blood and lymphatic system disorder	bradycardia	1 (0.8) c	--
	chronic renal insufficiency	1 (0.8) k	--
	circulatory instability (t)	1 (0.8) z	--
	hemorrhage (c, t)	1 (0.8) i	--
	neutropenia (c)	1 (0.8) p	--
	thrombocytopenia	1 (0.8) j	--
	syncope (t)	1 (0.8) c	--
	dyspnea (c, t)	1 (0.8) v	1 (0.5) a
	anemia (c, t)	3 (2.4) г, j, j	1 (0.5) l
	dyspnea (c, t)	1 (0.8) v	1 (0.5) a
	agranulocytosis	--	1 (0.5) l
	burning sensation (v)	--	1 (0.5) x
	neutropenia (c, t)	--	1 (0.5) l
	absolute arrhythmia	--	1 (0.5) д
congenital, familial and genetic disorders	hypersensitivity (c, t, v)	1 (0.8) л	1 (0.5) u
endocrine disorders	exsiccosis	1 (0.8) г	--
gastrointestinal disorders	dysphagia	1 (0.8) г	--
	nausea (c,t,v)	3 (2.4) h, б, и	--
	vomiting (c,t,v)	3 (2.4) r, б, и	--
	diarrhea (c, t, v)	--	1 (0.5) b
	stomatitis (c, t)	--	1 (0.5) l
general disorders and administration site conditions	discomfort	1 (0.8) c	--
	loss of appetite (c, t)	1 (0.8) h	--
	chills (c, t, v)	2 (1.6) d, j	1 (0.5) н
	pain (c, t, v)	3 (2.4) h, I, y	1 (0.5) f
	pyrexia (c, t, v)	1 (0.8) v	4 (2.2) s, u, f, н
	cachexia	--	1 (0.5) l
	edema (v)	--	2 (1.1) l, o
	induration (v)	--	1 (0.5) x
	local reaction (c, t, v)	--	1 (0.5) f
	swelling (c, t, v)	--	1 (0.5) o
hepatobiliary disorders	ascites	--	1 (0.5) g
immune system disorder	polyneuropathy (c)	--	1 (0.5) o
infections and infestations	sepsis	1 (0.8) q	1 (0.5) l
metabolism and nutrition disorders	hypocalcemia	1 (0.8) h	1 (0.5) l
	hypokalemia	--	1 (0.5) l
musculoskeletal and connective tissue disorders	Dupuyrtren’s contracture	1 (0.8) q	--
	hypotonia	1 (0.8) c	--
nervous system disorders	Vertigo (c, t)	1 (0.8) h	--
psychiatric disorders	reduced general condition	1 (0.8) y	4 (2.2) g, m, n, w
renal and urinary disorders	renal insufficiency	--	1 (0.5) m
	urinary retention	--	1 (0.5) i
respiratory, thoracic and mediastinal disorders	cough	1 (0.8) r	--
	atelectasis	--	1 (0.5) e
	pleural effusion	--	1 (0.5) l
skin and subcutaneous tissue disorder	erythema (c, t, v)	1 (0.8) з	2 (1.1) t, x
vascular disorder	hemorrhoids (com)	1 (0.8) k	--
	thrombosis (t)	--	1 (0.5) m
Not specified	AE, unspecified	1 (0.8) ж	--
AE frequency, *n* (%)		38 (30.2) ^1)^	37 (20.1) ^1)^

Adverse events per treatment group classified as MedDRA (MedDRA Version 20.1) preferred terms and grouped by System Organ Class. As causality was not attributed to adverse events (AEs) in the present study and because AEs could have been reactions to concomitantly applied agents, expected AEs according to clinical studies and Summary of Product Characteristics (SmPCs) have been indicated: t for targeted therapy including monoclonal antibodies (mAbs), tyrosine kinase inhibitors (TKIs), and immune checkpoint inhibitors (ICIs), c for chemotherapy, and v for VA therapy; **^1)^**comparison of AE frequency combinational vs. control: χ^2^ = 4.119, *p*-value = 0.042. Each alphabetical letter (a, b, etc.) in the right two columns represents a different patient having experienced AEs. --, not applicable.

## Abbreviations

AE, adverse event; CTx, chemotherapy; ICH, International Council for Harmonisation of Technical Requirements for Pharmaceuticals for Human Use; ICI, immune checkpoint inhibitor; IO, integrative oncology; IQR, interquartile range; MedDRA, Medical Dictionary for Regulatory Activities; mAb, monoclonal antibody; NO, network oncology; i.v., intravenous; s.c., subcutaneous; SmPC, Summary of Product Characteristics; TKI, tyrosine kinase inhibitor; UICC, Union for International Cancer Care; VA, *Viscum album* L.
